# Use of a clinical decision support system to increase osteoporosis screening

**DOI:** 10.1111/j.1365-2753.2010.01528.x

**Published:** 2012-02

**Authors:** Ramona S DeJesus, Kurt B Angstman, Rebecca Kesman, Robert J Stroebel, Matthew E Bernard, Sidna M Scheitel, Vicki L Hunt, Ahmed S Rahman, Rajeev Chaudhry

**Affiliations:** 1 Assistant Professor, Center for Innovation, Mayo ClinicRochester, Minnesota, USA; 2 Instructor, Division of Primary Care Internal Medicine, Center for Innovation, Mayo ClinicRochester, Minnesota, USA; 3 Assistant Professor, Department of Family Medicine, Center for Innovation, Mayo ClinicRochester, Minnesota, USA; 4 Senior Information Research Analyst, Center for Innovation, Mayo ClinicRochester, Minnesota, USA

**Keywords:** clinical decision support system, delivery of health care, mass screening, osteoporosis, technology

## Abstract

**Background:**

In 2002, the US Preventive Services Task Force recommended routine osteoporosis screening for women aged 65 years or older. However, studies have indicated that osteoporosis remains underdiagnosed, and various methods such as the use of health information technology have been tried to increase screening rates. We investigated whether we could boost the low rates of bone mineral density testing with implementation of a point-of-care clinical decision support system in our primary care practice.

**Methods:**

We retrospectively reviewed the medical records of female patients eligible for osteoporosis screening who had no prior bone mineral density test who were seen at our primary care practice sites in 2007 or 2008 (before and after implementation of a point-of-care clinical decision support system).

**Results:**

Overall, screening rates were 80.1% in 2007 and 84.1% in 2008 (*P* < 0.001). Of patients who did not have osteoporosis screening before the visit, 5.87% completed the screening after the visit in 2007, compared with 9.79% in 2008 (when the clinical support system was implemented), a 66.7% improvement (*P* = 0.025).

**Conclusion:**

Clinical decision support for primary care doctors significantly improved osteoporosis screening rates among eligible women. Carefully designed clinical decision support systems can optimize care delivery, ensuring that important preventive services such as osteoporosis screening for patients at risk for fracture are performed while unnecessary testing is avoided.

## Introduction

Despite national osteoporosis screening guidelines recommending routine screening for women aged 65 years or older [1–3], studies have identified rates of screening in primary care settings as low as 12% [4]. Studies of the efficacy of different interventions to screen more eligible women has led to mounting evidence that health information technology (HIT) improves adherence to osteoporosis screening guidelines [5]. However, doctors have been reluctant adopters of HIT because of the expense and time required to change their office systems or because they are not convinced of the benefits of doing so [6]. As a result, the health care industry lags behind other sectors in IT investment [7]. This means that applications that may narrow gaps in care (e.g. clinical decision support systems that prompt doctors to discuss preventive services with patients) are not being fully deployed.

Osteoporosis, a common condition that affects about eight million women and two million men in the USA, is characterized by low bone mass and structural deterioration of bone tissue, leading to increased bone fragility. It poses an increased risk of fracture for people older than 50 years and, if left untreated, its debilitating consequences constitute a substantial national economic burden [1,8]. Experts predict that total medical costs from fractures will rise by almost 50%, to $25.3 billion, in 2025 (from $19 billion in 2005) [1,9]. To address this growing threat, the 2002 US Preventive Services Task Force, the National Osteoporosis Foundation and, most recently, the American College of Preventive Medicine, recommended routine screening for osteoporosis for all women aged 65 years or older [1–3]. Screening should begin earlier in postmenopausal women with risk factors for osteoporosis [1,3]. To date, mineral bone density measured by dual-energy X-ray absorptiometry (DEXA) is the best predictor for fracture [1,2,10].

However, despite these recommendations, the osteoporosis screening rate remains low. One 2002 study found that only 12–34% of women at high risk for fracture in a managed care network were screened for osteoporosis [4]. Another study reported that just 45% of 6311 at-risk patients seen by 160 doctors in 10 primary care sites had a prior bone mineral density test [11]. The mean rate of screening in one primary care group practice was 56% [12]. Less than 50% of 1200 adults aged 60 years or older surveyed in the north-eastern USA said that their doctor recommended osteoporosis screening [13].

Time constraints are a major limiting factor in primary care doctors' ability to deliver preventive services [14]. Clinical decision support technology increases efficiency and the likelihood that patients will get the care they need. Such information systems help identify and screen patients who are due for preventive services, independent of direct doctor contact. A review of randomized controlled trials of the use of computerized prompts for the provider at the point of care found an increased rate of bone mineral density testing [15].

In a prior study at Mayo Clinic Rochester, a population-based information system improved the rate of mammography screening [16]. A subsequent study from the same institution showed significant improvement in osteoporosis screening with the use of a medical record-based information system. In that study, women eligible for osteoporosis screening were identified and classified into either an intervention group that was sent letters requesting them to make an appointment for a DEXA scan or to a control group [17]. Although limited by the small number of patients who underwent initial osteoporosis screening, the study still showed very encouraging results, as 25% of the intervention group completed screening in response to one letter inviting them to the screening. Another recently completed study using a clinical decision support system showed improved delivery of Abdominal Aortic Aneurysm screening (R. Chaudhry *et al*., unpublished observation). In this study, we looked at improving the osteoporosis screening rate of eligible female patients seen at the Primary Care Internal Medicine and Family Medicine clinics through implementation of a point-of-care clinical decision support system in January 2008.

## Methods

Mayo Clinic's Employee and Community Health practice provides primary care to 140 000 patients in Primary Care Internal Medicine, Family Medicine and Community Pediatrics. The Employee and Community Health practice sites in the Rochester area are staffed by 45 internists and 40 family doctors.

The Generic Disease Management Systems (GDMS) software is a Web-based application developed by VitalHealth, a joint venture between Mayo Clinic and Netherlands-based Noaber Foundation. Details of its features were reported in a previous study (R. Chaudhry *et al*., unpublished observation). The GDMS includes a rules-based application coded with national guidelines for age- and sex-specific preventive services and process and outcome measures for diabetes and coronary artery disease. On the basis of the data from Web services, the rules provide point-of-care decision support regarding the services that the patient needs at the time of their visit and in the next 90 days. It also automatically checks for a prior bone density test.

The GDMS was developed and successfully pilot tested in December 2007, and the system was made available to all practice sites of the Employee and Community Health practice for adults in January 2008. With the new workflow, when a patient visits a site for any reason (e.g. acute condition, chronic disease, annual examination), a paper copy of the GDMS summary screen is printed by check-in staff and is included in the rooming packet for the allied health staff. Women who meet initial osteoporosis screening criteria (65 years or older) can then have the order placed (per protocol) for DEXA by the rooming personnel. The order is then activated by the provider after discussion with the patient.

An independent data abstractor reviewed all the records of female patients aged 65 years or older who were seen in three Employee and Community Health practice sites in 2007 and 2008 and who had no prior bone density test. The completion rate of initial osteoporosis screening from January to December 2007 was compared with the rate from January to December 2008 (1 year after the GDMS implementation). A subanalysis of osteoporosis screening completion within 30 days from the clinic visit was done for the first 4 months of 2007 and 2008. This was undertaken to evaluate the efficiency of the new workflow and the point-of-care clinical decision support tool in facilitating screening completion. Chi-squared test was used to determine differences in outcome and a two-tailed *P*-value of <0.05 was considered statistically significant.

## Results

In 2007, 7263 women were eligible for osteoporosis screening. Of these women, 5817 (80.1%) completed an initial screening test by the end of the year. After implementation of the point-of-care clinical decision support tool in 2008, 6234 (84.1%) of the 7411 eligible women completed osteoporosis screening by the end of the year (see Table 1; χ^2^ = 40.254; *P* < 0.001).

**Table 1:** Women obtaining osteoporosis screening before and after clinical decision support tool implementation

	2007 (before clinical decision support tool implementation)	2008 (after clinical decision support tool implementation)
Number of women eligible for screening	7263	7411
Number of women screened	5817	6234
Percentage of eligible women screened	80.1%	84.1%

Subanalysis of data abstracted on osteoporosis screening among Family Medicine practice sites during the first 4 months of the GDMS implementation in 2008 was done (see Table 2); the resulting data were compared with data from the first 4 months in 2007. Of the 2212 eligible women seen at those sites from January to April 2007, 545 had no baseline osteoporosis screening; only 32 (5.87%) screenings were completed after a clinic visit that year. During the first 4 months of GDMS implementation in 2008, 490 of the 2418 eligible women seen in were identified by the GDMS as requiring baseline osteoporosis screening. Of these women, 48 (9.79%) completed screening within 30 days after the visit (*P* = 0.025).

**Table 2:** Subanalysis of osteoporosis screening data among Family Medicine practice sites during first 4 months of implementation

	2007 (January–April)	2008 (January–April)
Number of women eligible for screening	2212	2418
Number of women with no baseline screening	545	490
Number of women screened	32*	48†
Percentage of eligible women screened	5.87%	9.79%

* During 2007.

† Within 30 days of clinic visit.

## Discussion

In our study, the overall osteoporosis screening rate among all eligible female patients grew roughly 4% 1 year after initiation of a point-of-care clinical decision support tool (i.e. the GDMS) in primary care. The tool's utilization in face-to-face encounters led to increased completion of screening after a clinic visit by identifying eligible patients and alerting their providers, who then engaged patients in the discussion. This was clearly shown by the 66.7% improvement for the eligible patients compared with the same time period in 2007, when we did not have the decision support tool (*P* = 0.025). However, not all patients who were identified by the decision support to be eligible for screening received it, which highlights the importance of having processes in place to support the decision support for the services that are due. Primary care doctors have many tasks to perform during a 15- to 20-minute visit; having allied health staff take on the responsibility for helping the doctors deliver the necessary services can lead to better results [18].

Improvement of health care value relies on payment reform that pays for value rather than volume of services. Decision support tools that help improve quality are essential. Without these tools, health care providers would need to determine the need for screening through a manual process, which is time-consuming and unreliable. Also, identifying women in need of treatment through screening can markedly reduce fractures, improve quality life and contain overall health care costs on an individual and national basis [19,20].

Our study has several strengths, including its large sample size and completeness in identifying women for screening with the GDMS. We showed significant improvement in osteoporosis screening completion among a study population of community-dwelling women seen in a primary care practice, an area that is most affected by performance measures on preventive health screening and chronic disease management. The use of point-of-care clinical decision support tools like the GDMS can help increase primary care providers' efficiency of time allocation, enable them to engage patients in their care and lead to improved outcomes and higher career satisfaction by decompressing taxed practices [5,21]. Our results cannot be generalized to all minority groups, because most of the women in our study are White. However, osteoporosis is most prevalent in White and Asian women [1].

In conclusion, we observed an improved baseline osteoporosis screening rate among eligible female patients in our primary care practice using the GDMS point-of-care clinical decision support tool. Early identification of at-risk patients allows the institution of non-pharmacologic and pharmacologic measures that can help prevent devastating and potentially life-threatening fractures. This is an important step towards achieving better health outcomes and may serve as a model for future public health initiatives.

We may be able to expand the reach of our osteoporosis screening programme even more by developing a deeper understanding of workflow, information flow, barriers to use and provider needs in our primary care clinics [22]. Such knowledge could help us optimize use of the clinical decision support tool to ensure that patients get the care they need when they need it.

## Conflict of interest

Dr Chaudhry is employee of Mayo Clinic and the inventor of GDMS referenced in this publication. Mayo Clinic has licensed this technology to a commercial entity (VitalHealth Software) but to date, has received no royalties. Dr Chaudhry receives no royalties from the licensing of this technology.
